# Silencing circFTO inhibits malignant phenotype through modulating DUSP4 expression in clear cell renal cell carcinoma

**DOI:** 10.1038/s41420-022-01138-7

**Published:** 2022-09-20

**Authors:** Chen Yang, Yiwen Zang, Siqi Wu, Quan Zhou, Yuxi Ou, Qiang Ding, Hao Wang, Zuquan Xiong

**Affiliations:** 1 Huashan Hospital, Fudan University, Shanghai, China; 2 Shanghai Medical College, Fudan University, Shanghai, China; 3 Fudan Institute of Urology, Huashan Hospital, Fudan University, Shanghai, China

**Keywords:** Renal cancer, Non-coding RNAs

## Abstract

Clear cell renal cell carcinoma (ccRCC) is the most diagnosed malignancy in kidney. Studies on the role of circular RNAs in kidney cancer are increasing. In this study, we employed high throughput sequencing and tissue micro array to detect and verify one of the key circular RNAs, circFTO, in ccRCC. The effect of circFTO on the proliferation and invasiveness of ccRCC cells and the corresponding mechanism were studied both in vitro and in vivo via multiple methods. We confirmed that circFTO was up regulated in ccRCC and correlated with a more aggressive phenotype. The up regulated circFTO could sponge and block the function of miR-514b-3p, a reported tumor suppressor, and caused overexpression of DUSP4. DUSP4 was found to lead to KRAS/ERK pathway activation, increased epithelial-mesenchymal transition (EMT) and inhibition of autophagy in ccRCC cells, which in the end boosted the proliferation and invasiveness of ccRCC. We thus concluded that circFTO/miR-514b-3p/DUSP4 axis may play an important role in ccRCC development and could be a potential biomarker and therapeutic target.

## Introduction

Renal cell carcinoma consists of a group of heterogenous cancers and is the third most diagnosed malignancy in urogenital system, among which clear cell renal cell carcinoma (ccRCC) as a major type [1, 2]. ncRNAs are RNAs that are not translated into proteins. The aberration of the complex interactions of ncRNAs are believed to contributed to many cancers [3]. It was not until 2012 that circular RNAs (cirRNAs), once thought to be functionless byproducts of aberrant RNA splicing, were found to be widespread in mammalian cells and have essential functions [3, 4].

CircRNAs are generated by back-splicing of pre-mRNAs. Due to the closed ring structure, circRNAs are much more stable than linear RNA. The regulatory mechanism of circRNAs as miRNAs sponges, known as ceRNA, has been well established since the work of Hansen et al. and Memczak et al. [4, 5]. MiRNAs are another group of ncRNAs which were much more studied compare with circRNAs. By base pairing to mRNAs, miRNAs can repress translation of mRNAs and down regulate specific gene expression, which is the main regulatory mechanism downstream of circRNA as ceRNA regulation. During the past decades, the aberrant expressions of many miRNAs have been found in many human cancers with distinct regulatory effects through ceRNA regulation [6].

In this study, we identified a novel circRNA, circFTO, was up-regulated in cancer tissue of ccRCC and higher expression of circFTO was associated with worse prognosis. Subsequently, we discovered that circFTO and DUSP4, a member of dual specificity phosphate (DUSP), both possess binding sites of miR-514b-3p. Up-regulation of circFTO might promotes the expression of DUSP4 and the proliferation and invasion ability of ccRCC cells. Mechanistically, we also elucidated the circFTO exerted biological consequence through increasing DUSP4 expression mediated ERK pathway.

## Results

### CircFTO is up regulated in ccRCC

**Fig. 1:**
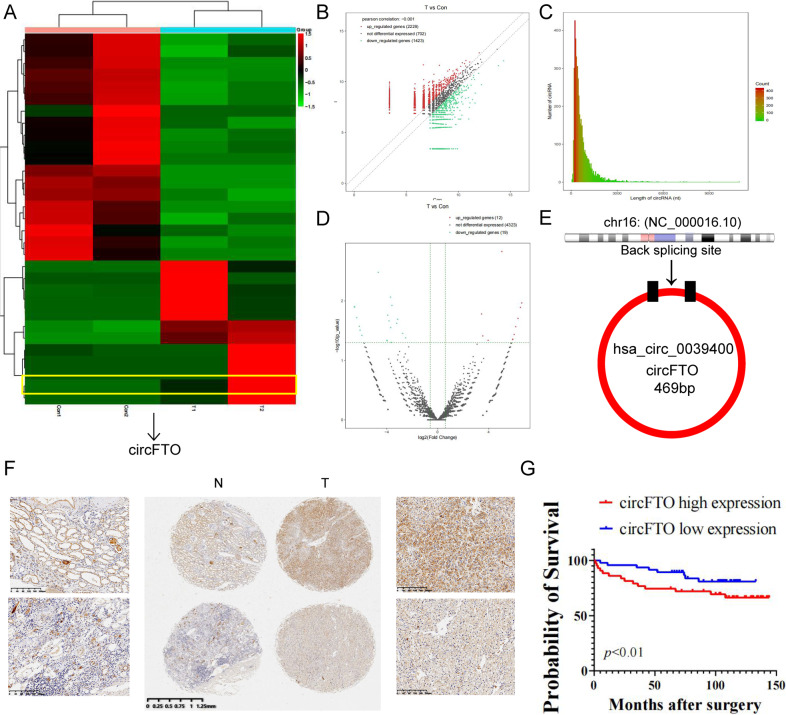
circFTO is up regulated in ccRCC and correlated with worse prognosis. **A** Heatmap of significant differential expressed circRNAs between tumor and normal tissue in ccRCC. **B** Scatterplots of differential expressed circRNAs between tumor and normal tissue. **C** Distribution of length of identified circRNAs. **D** Vocano plot of significant differential expressed circRNAs. **E** Genomic loci of FTO gene and circFTO. **F** Expression of circFTO in tumor and normal tissue identified by RNA in situ hybridization (ISH). **G** Prognostic significance of circFTO expression for ccRCC patients determined with ISH values. Median value was used as the cutoff.

We used high-throughput sequencing to look for differential expressed circRNAs between 2 pairs of ccRCC tissue and adjacent benign tissue (Fig. 1A) and a total of 2229 up re-gulated circRNAs and 1423 down-regulated circRNAs were identified in tumor tissue (Fig. 1B). Most of the circRNAs identified were less than 1500nt (Fig. 1C). 19 down-regulated and 12 up-regulated circRNAs were selected using the cutoff standard of p < 0.05 and |log2FC | > 2 (Fig. 1D). CircFTO, also known as hsa_circ_0039400, was one of the up-regulated hits. It is a 469nt circRNA derived from the FTO gene with a unique back-splicing site other than its lineal counterpart (Fig. 1E). As expected, we found that circFTO was more resistant to Rnase R treatment than its linear counterpart (Supplemtary Fig. 1A). Furthermore, Actinomycin D treatment indicated circFTO was more stable compared to its linear form (Supplemtary Fig. 1B, C). Then we performed ISH of circFTO in our TMA consist of 90 pairs of ccRCC and benign tissues. It was clear that circFTO was mainly located in cytoplasm and expressed more in tumor tissue (Fig. 1F). In addition, high circFTO expression was correlated with worse prognosis of ccRCC in our cohort (Fig. 1G).

### Knocking down circFTO suppresses ccRCC cells proliferation and invasiveness in vitro and in vivo

**Fig. 2:**
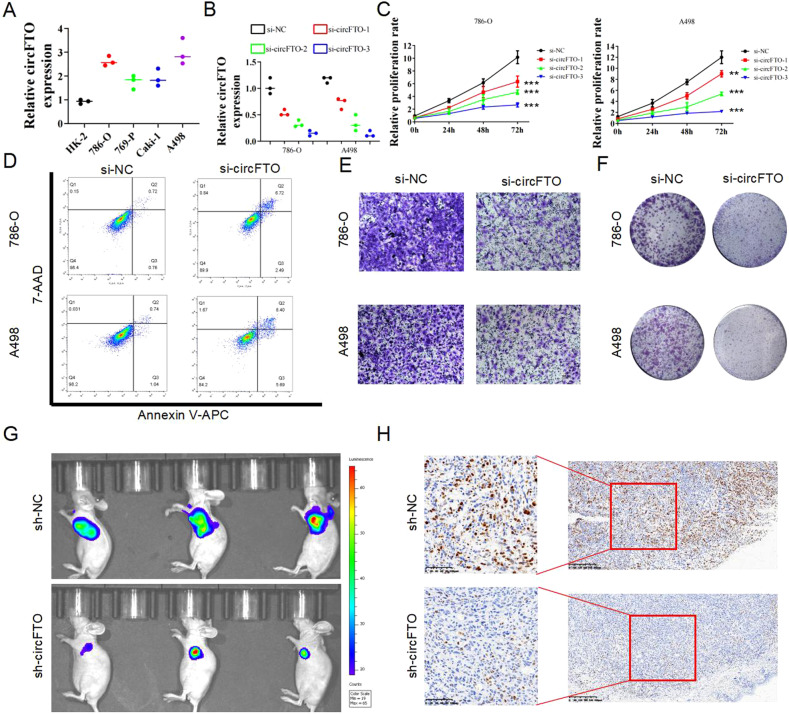
circFTO silencing suppresses ccRCC cells proliferation and invasiveness in vitro and in vivo. **A** Relative expression of circFTO in four renal carcinoma cell lines (786-O, 769-P, Caki-1 and A498) and the immortalized normal kidney cell line HK-2 identified with qRT-PCR. **B** Relative expression of circFTO in 786-O and A498 cells after transfected with si-circFTO with qRT-PCR. **C** CCK-8 assay, (**D**). apoptosis assay, (**E**) transwell assay and (**F**) colony formation assay of 786-O and A498 cells after transfected with si-circFTO and negative control. **G** Subcutaneous xenografts of sh-NC and sh-circFTO luciferase (luc)-labeled 786-O cells identified with live imaging. **H** Ki-67 staining of the eviscerated tumors.

Consistent with what was seen in clinical samples, the expression of circFTO in four renal carcinoma cell lines (786-O, 769-P, Caki-1 and A498) is higher than in the immortalized normal kidney cell line HK-2 (Fig. 2A). We designed 3 different small interfering RNAs (siRNAs) targeting circFTO (si-circFTO-1 to 3) and transfected them to 786-O and A498 cell lines. Each was able to decrease the expression of circFTO to certain level compared to the negative control (si-NC) (Fig. 2B) without decreasing the expression of FTO expression (Supplemtary Fig. 1D, E). Consistently, the more effective the siRNA was, the more it was able to impair the proliferation of transfected cells (Fig. 2C). Since si-circFTO 3 was the most effective one, we used it in all the following experiments. Cell apoptosis assay detected by flowcytometry showed that knock down of circFTO increased the rate of apoptosis in ccRCC cells (Fig. 2D). The invasion and colony formation ability were also decreased in circFTO knockdown cells, as detected by Transwell assay and colony formation assay (Fig. 2E, F). Furthermore, we injected luciferase (luc)-labeled 786-O cells transfected with si-circFTO and si-NC into mice to induce subcutaneous xenograft formation. Live imaging after 3 weeks showed a reduction of visible 786-O xenograft after knockdown of cirFTO (Fig. 2G). Ki-67 staining of the eviscerated tumors confirmed the down regulation of proliferation ability in si-circFTO groups (Fig. 2H).

### CircFTO serves as the RNA sponge for miR-514b-3p

**Fig. 3:**
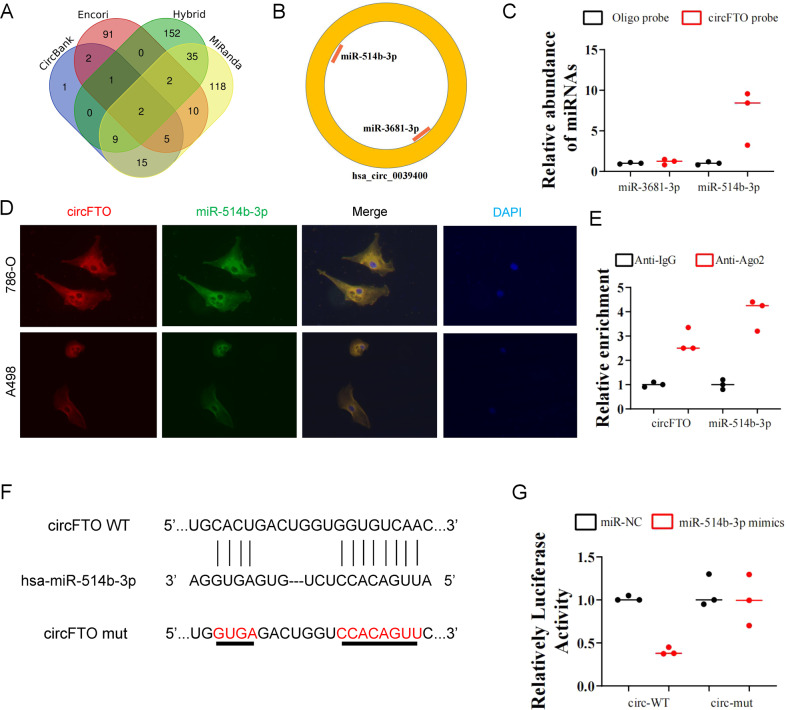
circFTO serves as the RNA sponge for miR-514b-3p. **A** and **B** Prediction of the potential target miRNAs of circFTO with four different datasets circBank, Encori, Hybrid and MiRanda. **C** RNA pulldown of miR-514-3p and miR-3681-3p in 786-O cells with Oligo or circFTO probe. Relative miRNA expression was detected by qRT-PCR. **D** Subcellular location of circFTO and miR-514b-3p detected by fluorescence in situ hybridization (FISH). **E** RIP against IgG or Ago2 in 786-O cells. Precipitated circFTO and miR-514-3p were detected by qRT-PCR. **F** The designs of dual-luciferase reporter vector of wild-type (WT) and mutated (Mut) circFTO sequences with binding site to miR-514b-3p. **G** Relative luc activity after transfection with miR-514b-3p mimic/negative control or with the circFTO WT/Mut in HEK293T cells.

The mostly studied function of circRNAs is their role as miRNAs sponges. To test whether circFTO can also work in this way, we first predict the potential target miRNAs of circFTO combing four different datasets circBank, Encori, Hybrid and MiRanda (Fig. 3A, B). Among the two predicted miRNAs, only miR-514b-3p could be pulled down by the circFTO probe (Fig. 3C). miR-514b-3p was reported as a tumor suppressor in both esophageal carcinoma and colorectal cancer [7, 8]. To further confirm the interaction between circFTO and miR-514b-3p, we firstly performed RNA FISH. The results demonstrated the co-locolization of circFTO and miR-514b-3p in cytoplasm (Fig. 3D). Then Ago2 RNA immunoprecipitation (RIP) was applied for further validation and higher circFTO and miR-514b-3p levels were seen in anti-Ago2 RIP than in anti-IgG RIP (Fig. 3E). We also constructed dual-luciferase reporter vector of both wild-type (WT) and mutated (Mut) circFTO sequences and transfected them to HEK293T cells that contained miR-514b-3p mimics or the negative control (miR-NC) to confirm the binding of miR-514b-3p to circFTO (Fig. 3F). The dual-luciferase assays revealed that miR-514b-3p suppressed luciferase activity only in cells transfected with WT vector but not Mut vector. Our results demonstrated that miR-514b-3p was the downstream target of circFTO (Fig. 3G).

### MiR-514b-3p represses the expression of DUSP4 mRNA under circFTO regulation

**Fig. 4:**
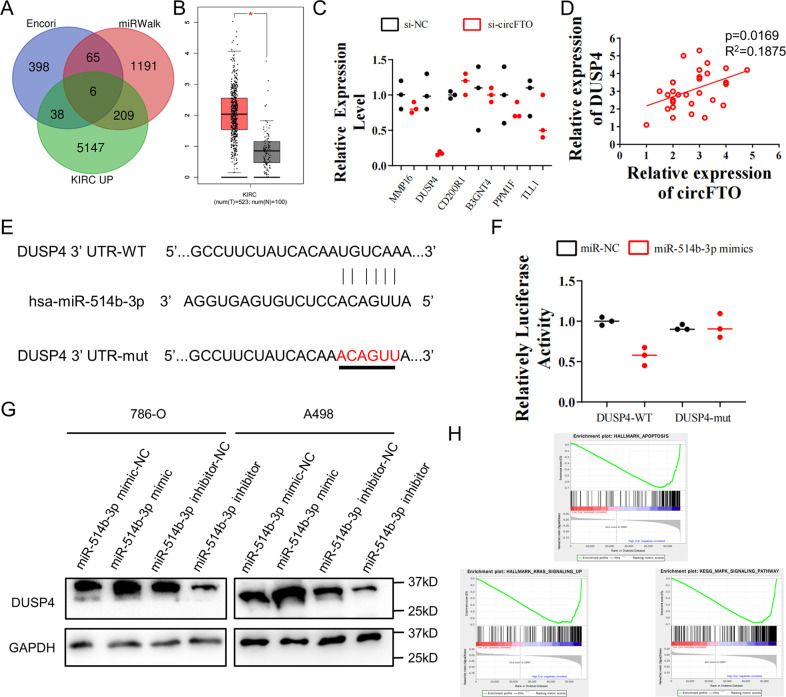
MiR-514b-3p represses the expression of DUSP4 mRNA. **A**. Prediction of the potential target mRNAs of miR-514b-3p. **B** Boxplot of DUSP4 expression in tumor and control in TCGA KIRC cohort. **C** Relative expression of 6 genes predicted to be target of miR-514b-3p in 786-O cells with/without knockdown of cricFTO. **D** Correlation of DUSP4 and circFTO expression in our clinical cohort. **E** The designs of dual-luciferase reporter vector of wild-type (WT) and mutated (Mut) DUSP4 sequences with binding site to miR-514b-3p. **F** Relative luc activity after transfection with miR-514b-3p mimic/negative control or with the DUSP4 WT/Mut in HEK293T cells. **G** DUSP4 level after transfected miR-514b-3p mimics/inhibitors and their control into 786-O and A498 cells detected by Western Blot. **H** Gene set enrichment analysis (GSEA) in TCGA KIRC cohort according to expression level of DUSP4. Median is used as cutoff.

We used Encori, miRWalk and differentially expressed gene in TCGA KIRC to predict the downstream target of miR-514b-3p (Fig. 4A, B). Six genes (MMP16, DUSP4, CD200R1, B3GNT4, PPM1F and TLL1) was identified and the expression of DUSP4 was the mostly suppressed gene when circFTO was knocked down in ccRCC (Fig. 4C). This result was strengthened by the positive correlation between DUSP4 and circFTO expression in clinical ccRCC cohort (Fig. 4D), thus we chose DUSP4 as the potential target of miR-514b-3p. To confirm their interaction, we constructed dual-luciferase reporter vector of both WT and Mut DUSP4 3ʹ UTR sequences. The dual-luciferase assays showed that miR-514b-3p suppressed luc activity only in cells transfected with WT but not Mut vector, indicating their direct interaction (Fig. 4E, F). Western Blot after transfected miR-514b-3p mimics/inhibitors into 786-O and A498 cells also showed a negative correlation between miR-514-3b and DUSP4 expression (Fig. 4G, Supplementary File 2). Since DUSP4 was indicated as one of the downstream targets of circFTO /miR-514b-3p, we preformed GSEA analysis in TCGA KIRC and identified MAPK, KRAS and apoptosis were mainly DUSP4 regulated pathways in ccRCC (Fig. 4H).

### circFTO promoted the proliferation and migration of ccRCC cells via miR-514-3b/DUSP4 axis in vitro

**Fig. 5:**
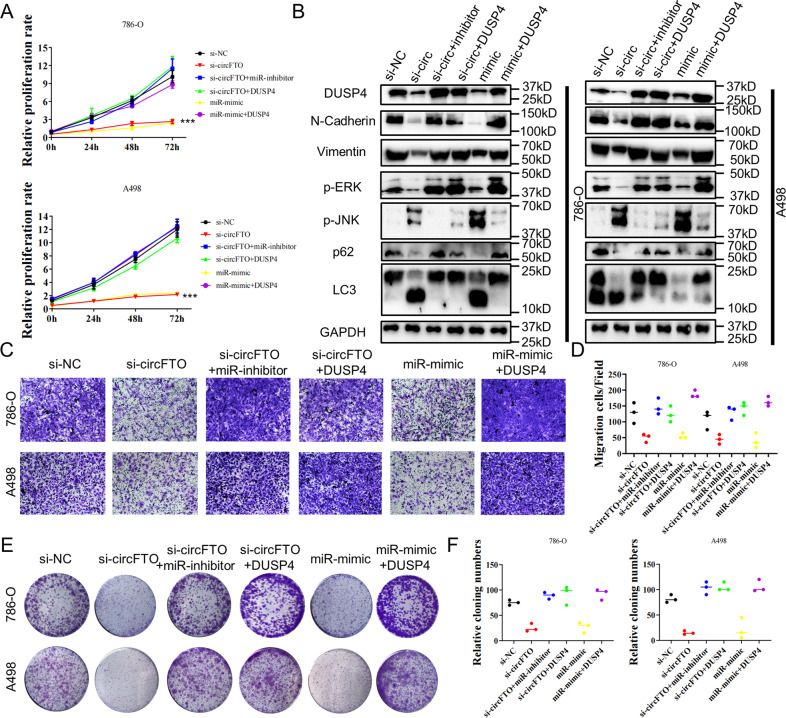
circFTO promoted the proliferation and migration of ccRCC cells via miR-514-3b/DUSP4 axis in vitro. **A** CCK8 assay were used to identify proliferation of si-NC/si-circFTO transfected 786-O and A498 cells with miR-mimic, miR-inhibitor and/or DUSP4 overexpression. **B** Western Blot results of core proteins level in si-NC/si-circFTO transfected 786-O and A498 cells with miR-mimic, miR-inhibitor and/or DUSP4 overexpression. **C** and **D** Transwell assay used to show the migration ability of si-NC/si-circFTO transfected 786-O and A498 cells with miR-mimic, miR-inhibitor and/or DUSP4 overexpression. **E** and **F** Colony formation assay were used to identify proliferation of si-NC/si-circFTO transfected 786-O and A498 cells with miR-mimic, miR-inhibitor and/or DUSP4 overexpression.

To confirm that circFTO affected the biological behavior of ccRCC cells by inhibit miR-514b-3p and boost the expression of DUSP4, we conducted CCK8 assay and colony formation assay to test the the effect on proliferation of 786-O and A498. Based on results of both experiments, we found that after silencing of circFTO or overexpression of miR-514b-3p, the proliferation ability of ccRCC cells were tremendously suppressed, and the suppression could be reversed by inhibition of miR-514b-3p and overexpression of DUSP4 (Fig. 5A, E, F). Similarly, transwell assay revealed that migration of ccRCC cells were impaired when circFTO was knocked down or miR-514b-3p was overexpressed but recovered when miR-514b-3p was inhibited or DUSP4 was overexpressed (Fig. 5C, D).

### circFTO activated KRAS/ERK pathway in ccRCC through miR-514-3b/DUSP4 axis

We performed gene set enrichment analysis (GSEA) in TCGA KIRC cohort to explore the possible function of DUSP4 in ccRCC. Cell apoptosis process, KRAS signaling and MAPK pathway were significantly enriched in DUSP4 highly expressed cases as we indicated previously (Fig. 4H). The RAS /mitogen-activated protein kinase (MAPK) pathway can play a central role in human cancer. Hyperactivated in different tumors, many of its elements are identified as oncogenes [9]. Besides, MAPK pathway has been reported to affect several other key biological process in cancer development including epithelial-mesenchymal transition (EMT) and autophagy [10, 11]. We then validated the function of DUSP4 in regulating MAPK pathway, EMT and autophagy. We found that after knocking down circFTO or overexpression of miR-514-3b, elevated expression of p-JNK and downregulation of p-ERK expression were observed in 786-O and A498 cells which indicates activation of ERK and suppression of JNK function in circFTO regulation. Parallelly, we saw changes that indicated decreased EMT (N-Cadherin and Vimentin) and increased autophagy (LC3 II/LC3I and p62). All these changes could be reverted by inhibiting the miR-514-3b or overexpression of DUSP4 (Fig. 5B, Supplementary File 2).

### circFTO knockdown and mTOR inhibition suppressed the proliferation and metastasis of ccRCC cells synergistically

**Fig. 6:**
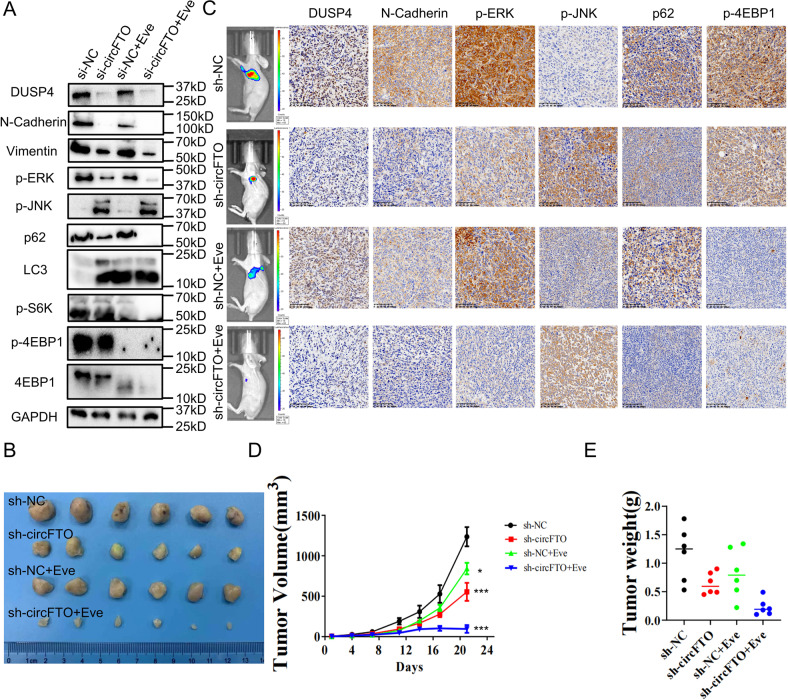
circFTO knockdown and mTOR inhibition suppressed the proliferation of ccRCC cells in vivo synergistically. **A** Western Blot results of core proteins level in si-NC/si-circFTO Western Blot results of core proteins level in si-NC/si-circFTO transfected 786-O cells co-treated with negative control/Everolimus. **B** Images of eviscerated tumors of subcutaneous xenografts of sh-NC and sh-circFTO 786-O cells co-treated with negative control/Everolimus. **C** IHC of the eviscerated tumors after sacrifice of mice confirmed the results from Western Blot. **D** and **E** Tumor volumes and weights were also shown.

**Fig. 7:**
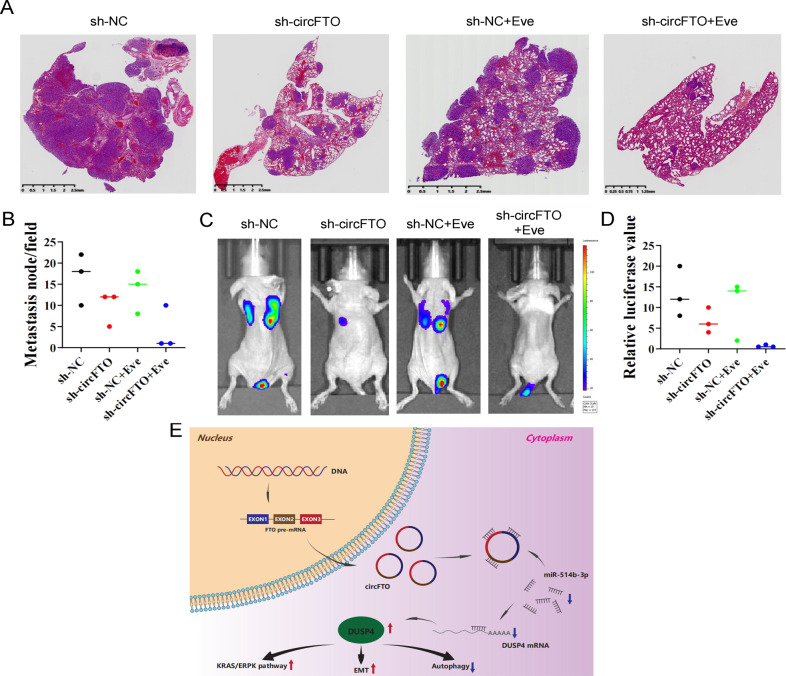
circFTO knockdown and mTOR inhibition suppressed the metastasis of ccRCC cells in vivo synergistically. **A** and **B** H&E staining showed the metastasis of sh-NC/sh-circFTO transfected 786-O cells to mice lungs co-treated with negative control/Everolimus. **C** and **D** Bioluminescence imaging detected the metastasis ability of si-NC and si-circFTO luciferase (luc)-labeled 786-O cells to lungs co-treated with negative control/Everolimus. **E** Schematic diagram of circFTO/miR-514b-3p/DUSP4 in ccRCC.

RAS/ERK pathway and mTOR pathways are two of the most important pathways that control the fate of cells. Interactions between these two pathways and co-regulation of downstream targets have been reported in previous research [12]. Inhibition of mTOR pathway would eventually lead to autophagy activation [13]. The relationship between autophagy dysregulation and cancers has been well established [14] as activation of autophagy in cancer cells can significantly decreased intracelluar large vacuoles and cell proliferation [15]. Since circFTO could impair the activity of ERK, we then tested whether a combination knockdown of circFTO and mTOR inhibition could futhermore restrict development of ccRCC. We firstly treated sh-NC and sh-circFTO 786-O cells with either control or Everolimus, a mTOR inhibitor and found that Everolimus not only suppressed the mTOR function, but also enhanced the ERK suppression and autophagy activation effect caused by circFTO knockdown (Fig. 6A, Supplementary File 2). To confirm this, we implanted luciferase (luc)-labeled sh-NC and sh-circFTO 786-O cells subcutaneously in nude mice and treated the mice with control or Everolimus.We founded that mTOR inhibition showed similar effect through IHC protein expression identification as we observed in vitro (Fig. 6C). What’s more, circFTO knockdown and mTOR inhibition showed a synergistical effect on controlling the tumor growth subcutaneously (Fig. 6B, D, E). To explore whether this combination treatment has an effect on tumor metastasis, we injected luc-labeled sh-NC and sh-circFTO 786-O cells intravenously into caudal veins of nude mice (Fig. 7A). Both H&E staining (Fig. 7B) and live imaging demonstrated that compared with sh-NC, sh-circFTO significantly reduced tumor metastasis into lung, and Everolimus treatment could augment this effect (Fig. 7C, D). Our results indicated circFTO knockdown and mTOR inhibition could suppress the proliferation and metastasis of ccRCC cells synergistically which give us a profound view of circFTO/miR-514-3b/DUSP4 axis in ccRCC (Fig. 7E).

## Discussion

With the development of next-generation sequencing, thousands of circRNAs have been identified, and their stable closed-ring structure and tissue-specific location endowed them with the ability to have widespread function in human tissues [16]. Located mainly in cytoplasm, circRNAs can function as miRNA sponges and interact with RNA-binding protein. Recently they have been found to regulate transcription or even been translated [17]. To date, miRNA sponges are the most studied regulatory mechanism of circRNA. In 2013, Hansen et al. reported that ciRS-7, a circRNA highly expressed in human brain, can serve as miRNA sponges for miR-7 [18] and many circRNAs since then were found to participate in cancer development via such mechanism [19–21], with ccRCC no exception [22]. In this study, we analyzed the circRNA expression profiles of 2 pairs of ccRCC tissue and the matched adjacent normal tissue, and we found that circFTO was up-regulated in cancer tissue and higher expression of circFTO was associated with worse prognosis. Furthermore, knocking down circFTO suppressed ccRCC cells’ proliferation and invasiveness in vitro and in vivo. FTO is well notorious for its role in obesity development and tumor occurrence. As a N6-methyladenosine (m6A) demethylase, it regulates cancer stem cell function, and promotes the growth, self-renewal and metastasis of cancer cells [23]. In this study we have not dug into the relationship between expression of FTO and circFTO in ccRCC, but it should worth further studying.

Though the function of miR-514p-3b in cancer has not been studied extensively, it was reported to a tumor suppressor in both esophageal carcinoma and colorectal cancer [7, 8] However, its role in ccRCC remains unclear. Indeed, in this study, we confirmed the overexpression of miR-514p-3b could repress the proliferation and invasiveness in ccRCC cells. Combining results from different databases, we predicted it as the target of circFTO, and this was proved by RIP, RNA pulldown and dual-luciferase assay.

As a member of dual specificity phosphate (DUSP), DUSP4 was reported to participate in cancer development from several aspects. DUSP4 is a promoter of EMT, and targeting DUSP4 can alleviate the EMT process and suppress tumor growth and drug resistance in breast and gastric cancer [24–27]. The other role of DUSP4 in cancer is MAPK pathway inhibitor, and DUSP4 inhibition can cause the activation of ERK pathway which is involved in tumor invasiveness and drug resistance in breast and pancreatic cancer [28–30]. DUSP4/MAPK pathway can also affect autophagy activation [31]. Here in this study, we observed that higher DUSP4 expression indicated EMT process promotion and suppression of autophagy. We also found that DUSP4 was related to KRAS/MAPK pathway via GSEA. However, in converse to several previous reports, we found a dual effect of DUSP4 on MAPK pathway, and that is the activation of ERK and inactivation of JNK. In general, we confirmed a tumor promoter role of DUSP4 in ccRCC as a downstream target of miR-514b-3p. Firstly, it was significantly downregulated in tumor tissue in TCGA KIRC cohort. Also, we observed an increase of tumor proliferation and invasion in DUSP4 overexpressed ccRCC cells in vitro and in vivo.

## Materials and methods

### Cell lines

The human immortalized normal kidney cell line HK-2 and renal carcinoma cell lines (786-O, 769-P, Caki-1 and A498) were purchased from the Type Culture Collection (Shanghai, China) at the Chinese Academy of Sciences. All cell lines were authenticated by STR profiling. HK-2 cells were cultured in K-SFM medium (Gibco, USA, 17005042). 786-O and 769-P cells were cultured in 1640 medium (Gibco, USA). Caki-1 cells were culured in McCoy’s 5 A (Modified) medium (Gibco, USA, 16600082). A498 cells were cultured in Dulbecco’s modified Eagle’s medium (Gibco, USA, 30030). All medium was supplemented with 10% fetal bovine serum (FBS) and 1% Penicillin-Streptomycin (Gibco, USA, 15070063). Cells were cultured at 37 °C in a humidified incubator with 5% CO_2_.

### Patient samples and sequencing

Ninety pairs of ccRCC tissue and adjacent normal tissue in a tissue microarray (TMA) were collected from 82 ccRCC patients who received partial or radical cystectomy in Huashan Hospital, Fudan University enrolled between 2008 and 2014 (Supplementary File 1, Table 1). The tissues were mostly deposited in liquid nitrogen or 4% paraformaldehyde solution for future experiments and the rest was each examined by two pathologists to confirm the diagnoses histologically and pathologically according to the 8th edition of the TNM classification of the Union for International Cancer Control (UICC, 2016). Written informed consent was acquired from each participant. The project was approved by the Board and Ethics Committee of Huashan Hospital, Fudan University (Approval number KY2011-009). Total RNA was extracted from 2 paired ccRCC/adjacent tissues with TRIzol (Invitrogen, USA). RNA-seq Library Prep Kit (Vazyme Biotech, China) was used with 3 μg of total RNA from each sample to erase ribosomal RNA and retain mRNA, miRNA and non-coding RNAs. Differential expressed circRNAs were further analyzed according to cutoff standard mentioned above (Supplementary File 3).

### RNA in situ hybridization (ISH)

CircFTO expression in ccRCC was determined via biotin-labeled circFTO probes. ccRCC TMA was deparaffinized with xylene and absolute ethanol, then incubated with biotin probes. DAB substrate was applied for the colorimetric detection of circFTO co-stained with hematoxylin and followed by dehydration.

### RNA Fluorescence in situ hybridization (FISH)

Specific probes to circFTO and probes against miR-514b-3p were prepared (Geneseed Biotech, Guangzhou, China). FISH were performed as previous studies [32].

### RNase R treatment

Total RNA extracted from 786-O and A498 cells was treated with RNase R (Epicenter Technologies, USA) for 30 min at 37 °C. Relative expression of circFTO and FTO mRNA was analyzed by qRT-PCR.

### Actinomycin D assays

786-O and A498 cells were seeded in six-well plates at a density of 500,000 cells per well. Two μg/ml Actinomycin D (Sigma) was added after 24 h and cells were harvested for qRT-PCR analysis.

### RNA extraction and qRT-PCR

Total RNA was extracted with TRIzol reagent (Invitrogen) according to the manufacturer’s instruction and cDNA was then synthesized by using PrimeScript^TM^RT Reagent Kit (TaKaRa, Japan). qPCR was performed on a CFX 96 real-time PCR system. The 2_DDCT method was used to normalize relative expressions of target genes with GAPDH as reference. Primers were shown in Supplementary File 1.

### Vector construction and cell transfection

Three siRNA (si-circFTO1 to 3), miR-514b-3p mimic and inhibitor were designed and cloned into the pGPU6/GFP/puromycin vector (GenePharma, Shanghai, China. See Supplementary File 1). Lentiviral packaging, infection and puromycin selection were similar to our previous work. Lipofectamine 2000 (Invitrogen, USA) was used to transfect these lentiviruses into 786-O and A498 cells [32].

### Luciferase reporter assay

Wild type and mutated cDNA fragments with predicted miRNA binding site of circFTO and DUSP4 3ʹ-UTR were amplified by PCR. Then fragments were recombined into psiCHECK-2 (Promega, Madison, WI, USA) were and co-transfected into HEK-293 cells with miRNA mimics or control mimics using Lipofectamine 2000 (Thermo Fisher Scientific, China). Cells were collected 48 h after co-transfection and luciferase activity was detected through dual-luciferase reporter assay kit (Promega, China). Relative luciferase activity was normalized to the Renilla luciferase internal control.

### Cell proliferation assay

Cell proliferation was determined by CCK8 assay and colony formation assay. For CCK-8 assay, 786-O and A498 cells were placed into 96-wells plates at a density of 1500 cells per well. OD450 was analyzed for each well at 0, 24, 48, and 72 h after seeding into the wells according to the manufacturer’s instructions. For the colony formation assay, transfected cells were seeded into 6-well plates at a density of 600 cells per well and maintained for 1 wee. Then the colonies were fixed and stained with 0.2% crystal violet for 15 min at room temperature. Cell colonies were counted and imaged.

### Apoptosis assay

Cell apoptosis was detected using the Annexin V-APC/7-AAD apoptosis kit (MultiSciences, China) following the manufacturer’s instructions. Briefly, cells were stained with Annexin-V-APC and 7-AAD for 30 min at room temperature in the dark and then subjected to a BD Accuri C6 flow cytometer (BD, USA). The cell apoptosis data were analyzed by Flowjo V10 software (Tree Star, San Francisco, CA, USA).

### Cell migration and invasion assay

For the wound healing assay, we placed 786-O and A498 cells into 6-well plates at a density of 20,000 cells per well. A wound was scratched with a 1,000 ml pipette tip after cells reaching monolayer and cell migrations at 0, 12, 24, 48, and 72 h were observed and imaged. For the Transwell assay, we used a BD Transwell chamber with 24 wells (Costar, USA) according to the manufacturer’s guidelines. After incubation for 24 h, the cells located on the upper surfaces of the transwell chambers were scraped and cells on the lower surfaces were fixed and stained by 0.2% crystal violet. The stained cells were photographed and counted in five randomly selected fields.

### Western blot analysis

Cells were lysed in ice-cold RIPA lysis buffer for proteins and equal amounts of proteins were separated using 10% SDS-PAGE. Then, proteins were transferred to nitrocellulose filter membrane membranes. The blots were blocked with freshly prepared 5% nonfat milk in PBST for 1 hours at room temperature and incubated at 4 °C overnight with primary antibodies against DUSP4 (Abcam, ab216576), p-ERK (CST, 4370), p-JNK (CST, 9255), p62 (Abcam, ab109012), LC3 (Abcam, ab192890), N-Cadherin (Abcam, ab76011), Vimentin (Proteintech, 10366-1-AP), p-mTOR (Abcam, ab109268), pS6K1 (CST, 9204), 4EBP1 (CST, 9644), p-4EBP1 (CST, 2855), GAPDH (Proteintech, 60004-1-lg) was detected as an endogenous control. After washing with PBST, the membranes were incubated with horseradish peroxidase-conjugated (HRP-conjugated) secondary antibodies at room temperature for 1 h. Signals were visualized with an ECL substrate (CLiNX, Shanghai, China) and captured using an ECL imaging system (CLiNX, Shanghai).

### RNA pulldown

Approximately 1 × 10^7^ ccRCC cells were collected and sonicated. Cell lysates were incubated with streptavidin-coupled magnetic beads (Life Technologies, USA) which were previously incubated with the biotin-labeled circFTO probes and oligo probes at 4 °C overnight for further experiments.

### RIP assay

The RIP assay was performed using a magnetic RIP RNA-binding protein immunoprecipitation kit (Millipore). Cells were harvested and lysed in lysis buffer and then incubated at 4 °C overnight with magnetic beads conjugated with an antibody against IgG or Ago2 (CST, 2897 S). Then the RNA binding to beads was purified using RNA extraction reagent after washing twice with wash buffer, and the relative enrichment abundance of circFTO and miR-514b-3p was detected by qRT-PCR.

### Animal study

For xenograft tumor model, three-week-old male BALB/c nude mice were divided randomly into two (for study of sh-circFTO) (*n* = 6) and four (for study of sh-circFTO and Everolimus) (*n* = 6) groups. A total of 2 × 10^7^ viable sh-NC or sh-circFTO 786-O cells were subcutaneously inoculated into the left flanks of nude mice. Everolimus was administrated 0.25 mg/kg three times a week p.o. Tumor growth was monitored every 5 days by measuring the width (W) and length (L) with calipers, and the volume (V) of the tumor was calculated using the formula V = (W^2 × L)/2. The tumors were excised and weighed when mice were sacrificed after 3 weeks.

For analysis of metastasis, luc vectors were transfected into sh-NC and sh-circFTO 786-O cells (2 × 10^5), and then the cells were tail-vein injected into 6 randomly divided mice and treated as with Everolimus like above. After 30 days, metastasis of 786-O cells was analyzed by bioluminescence imaging with intravenous injection of luciferin (200 mg luciferin/kg body weight) into the mouse tails and H&E staining of lungs tissue.

### Immunohistochemistry (IHC)

IHC was performed on formalin-fixed, paraffin-embedded tissue sectioned in 5 mm. The primary antibody used for IHC were the same as above. The immunostaining images were captured using an Axiophot light microscope (Carl Zeiss, Germany).

### Statistical analysis

Diffferences of data among groups were analyzed using the Student’s *t* test or one-way ANOVA. The Pearson’s correlation coefficient analysis was used to analyze the correlations. Kaplan–Meier curves and log rank tests were applied to evaluate the overall survival and analyzed by log-rank test. A *p* < 0.05 was considered to be statistically significant.

## Supplementary information

Supplementary File 1

Supplementary File 2

Supplementary File 3

## Data Availability

The datasets used and/or analyzed during the current study are available from the corresponding author on reasonable request.
