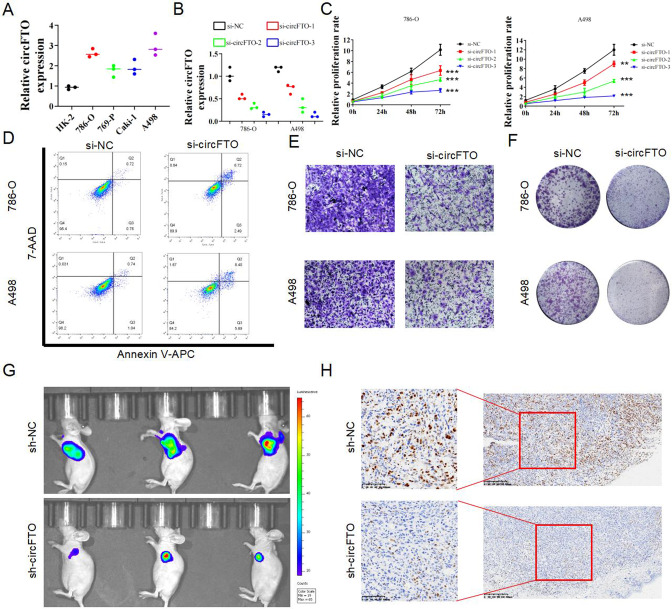# Correction: Silencing circFTO inhibits malignant phenotype through modulating DUSP4 expression in clear cell renal cell carcinoma

**DOI:** 10.1038/s41420-022-01279-9

**Published:** 2022-12-09

**Authors:** Chen Yang, Yiwen Zang, Siqi Wu, Quan Zhou, Yuxi Ou, Qiang Ding, Hao Wang, Zuquan Xiong

**Affiliations:** 1 Huashan Hospital, Fudan University, Shanghai, China; 2 Shanghai Medical College, Fudan University, Shanghai, China; 3 Fudan Institute of Urology, Huashan Hospital, Fudan University, Shanghai, China

**Keywords:** Renal cancer, Non-coding RNAs

Correction to: *Cell Death Discovery* 10.1038/s41420-022-01138-7, published online 20 September 2022

The original version of this article contained a mistake in Figure 2D. The authors apologize for the error. The correct figure can be found below. The original article has been corrected.