# Supplementary material for: Silencing circFTO inhibits malignant phenotype through modulating DUSP4 expression in clear cell renal cell carcinoma
Source: Cell Death Discov. 2022 Sep 20;8:392. doi: 10.1038/s41420-022-01138-7 (PMC9489864; doi:10.1038/s41420-022-01138-7)
Supplement: Supplementary file 1 [file 41420_2022_1138_MOESM1_ESM.pdf]

RT-qPCR primers:

GAPDH Forward	5-CAAGGCTGAGAACGGGAAG-3
GAPDH Reverse	5-TGAAGACGCCAGTGGACTC-3
FTO Forward	5-ACTTGGCTCCCTTATCTGACC-3
FTO Reverse	5-TGTGCAGTGTGAGAAAGGCTT-3
circFTO Forward	5-TCGGTGGGTGGAACTAAA-3
circFTO Reverse	5-GTGGAAGAAGATGGAGGGT-3
MMP16 Forward	5-AGCACTGGAAGACGGTTGG-3
MMP16 Reverse	5-CTCCGTTCCGCAGACTGTA-3
DUSP4 Forward	5-GGCGGCTATGAGAGGTTTTCC-3
DUSP4 Reverse	5-TGGTCGTGTAGTGGGGTCC-3
CD200R1 Forward	5-CAGAGGCATAGTGGTAACACCT-3
CD200R1 Reverse	5-GTGCCATTGCCCCAGTATTCT-3
B3GNT4 Forward	5-ACACAGTGTCTAGCGCCTCT-3
B3GNT4 Reverse	5-AAGGTATCCTTGGAACAGCCT-3
PPM1F Forward	5-GGGATTCCCAGGTCATTTTGG-3
PPM1F Reverse	5-TCCTGCCGTTCTGGTCTGT-3
TLL1 Forward	5-AACGAGCTGTGCTACAAGGTC-3
TLL1 Reverse	5-GCGTGGTCGATGAGGAAGA-3
miR-514b-3p	5-ACACTCCAGCTGGGATTGACACCTCTGTG-3
miR-3681-3p	5-ACACTCCAGCTGGGACACAGTGCTTCATCC-3
miRNA reverse primer	5-TGGTGTCGTGGAGTCG-3
U6 Forward	5-CTCGCTTCGGCAGCACA-3
U6 Reverse	5-AACGCTTCACGAATTTGCGT-3

Probes:

circFTO-Cy3:	5-TGGCATTGAGATCATCTGGCATGCCATTCT-3
circFTO-Bio:	5-TGGCATTGAGATCATCTGGCATGCCATTCT-3
miR-514b-3p-Dig:	5-TCCACTCACAGAGGTGTCAAT-3

Oligonucleotides:

si-NC sense	5-UUCUCCGAACGUGUCACGUTT-3
si-NC antisense	5-ACGUGACACGUUCGGAGAATT-3
si-circFTO-1 sense	5-AGAAUGGCAUGCCAGAUGATT-3
si-circFTO-1 antisense	5-UCAUCUGGCAUGCCAUUCUTT-3
si-circFTO-2 sense	5-AUGGCAUGCCAGAUGAUCUTT-3
si-circFTO-2 antisense	5-AGAUCAUCUGGCAUGCCAUTT-3
si-circFTO-3 sense	5-CAUGCCAGAUGAUCUCAAUTT-3
si-circFTO-3 antisense	5-AUUGAGAUCAUCUGGCAUGTT-3

Table 1 Relationship between the expression levels of circFTO and clinicopathological features in ccRCC

Characteristics	No. (%)	circFTO expression		
		High (%)	Low (%)	*P*-value
**Gender**				
Male	46 (56.1)	22 (47.8)	24 (52.2)	0.761
Female	36 (43.9)	16 (44.4)	20 (55.6)	
**Age**				
<60	35 (42.7)	16 (45.7)	19 (54.3)	0.922
≥60	47 (57.3)	22 (46.8)	25 (53.2)	
**Tumour size**				
<3 cm	33 (40.2)	10 (30.3)	23 (69.7)	0.017
≥3 cm	49 (50.8)	28 (57.1)	21 (42.9)	
**TNM stage**				
I	72 (87.8)	29 (40.3)	43 (59.7)	0.003
II-IV	10 (12.2)	9 (90.0)	1 (10.0)	
Total	82	38	44	

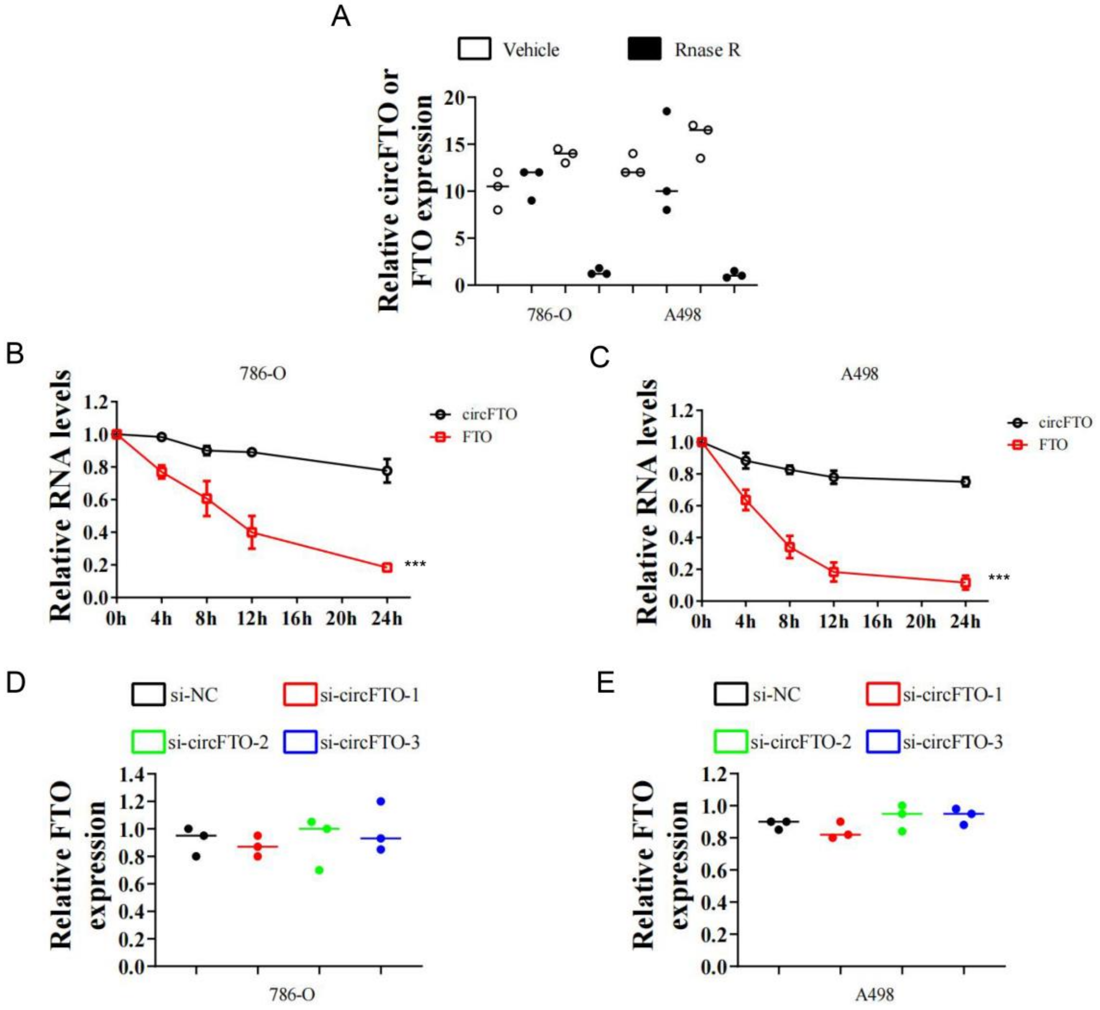

Supplementary Figure 1

A. Expression of circular and linear FTO in ccRCC cell lines treated with RNase R. **B. and C.** Relative expression of circFTO and linear FTO after actinomycin treatment in 786-O and A498 cell. **D. and E.** Relative expression of linear FTO after si-circFTO transfection in 786-O and A498
