# Supplementary material for: Silencing circFTO inhibits malignant phenotype through modulating DUSP4 expression in clear cell renal cell carcinoma
Source: Cell Death Discov. 2022 Sep 20;8:392. doi: 10.1038/s41420-022-01138-7 (PMC9489864; doi:10.1038/s41420-022-01138-7)

Original western blots in Figure 4

Figure4-DUSP4-1

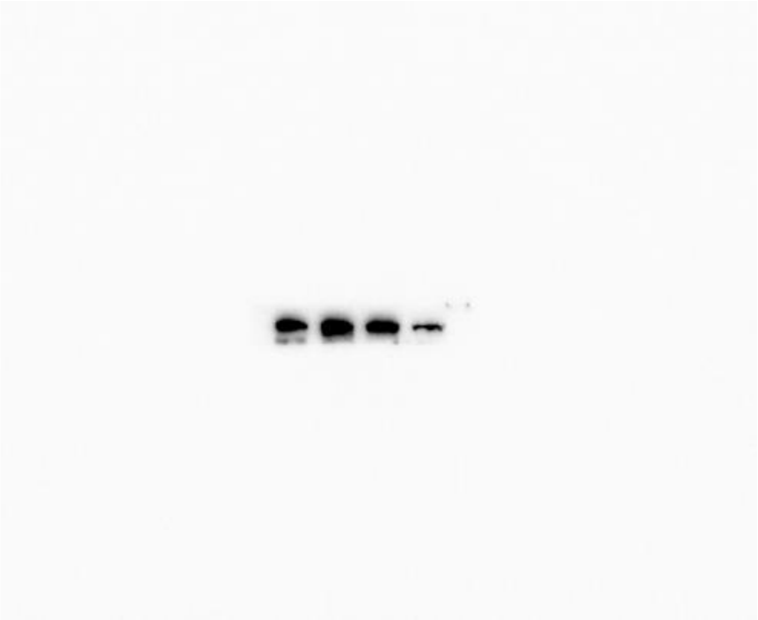

Figure4-DUSP4-2

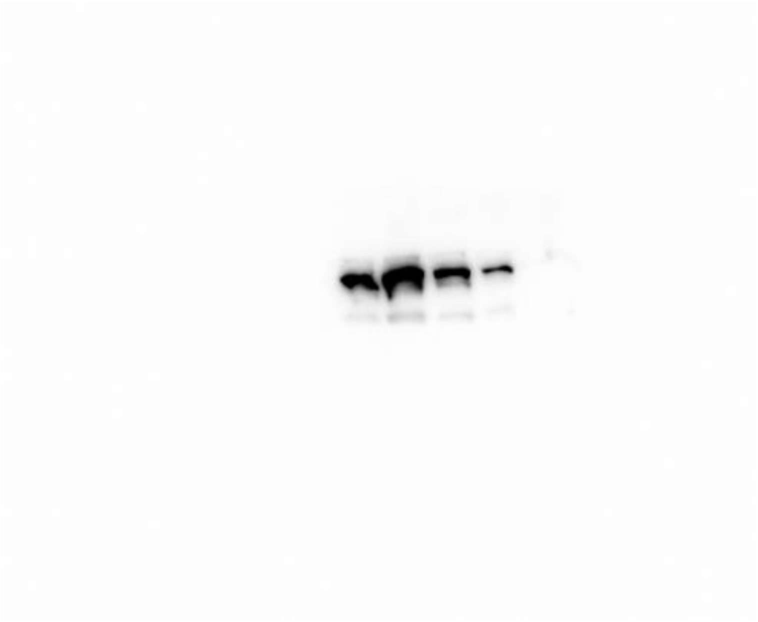

Figure4-GAPDH-1

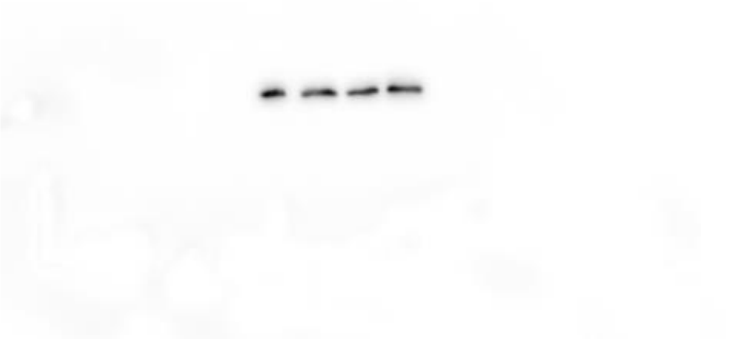
Figure4-GAPDH-2

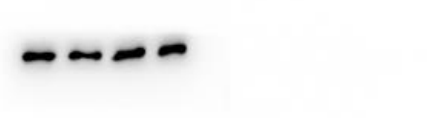

Original western blots in Figure 5

Figure5-DUSP4-1

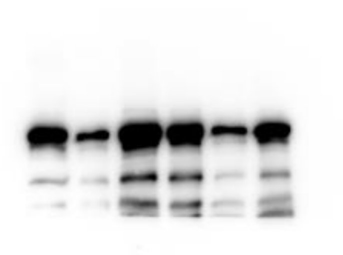

Figure5-DUSP4-2

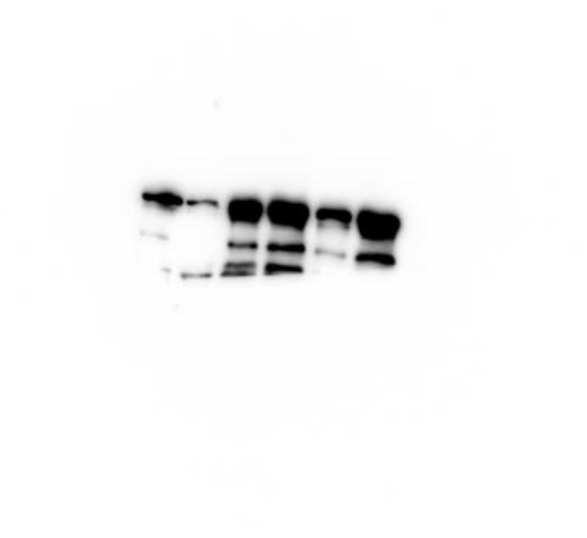

Figure5-GAPDH-1

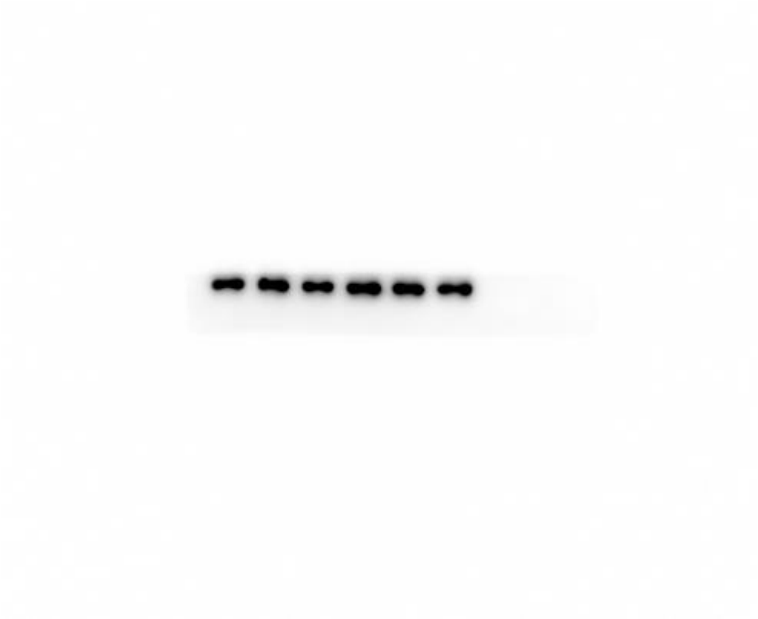

Figure5-GAPDH-2

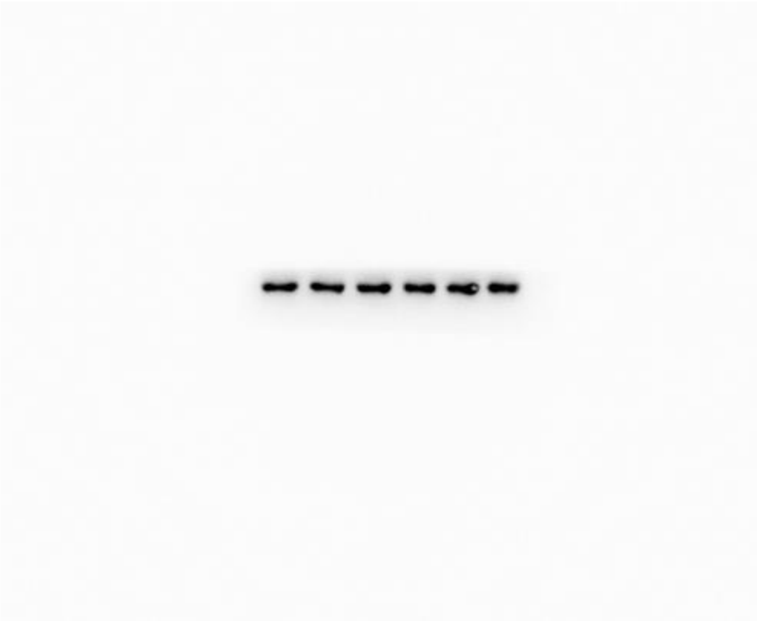

Figure5-LC3_1

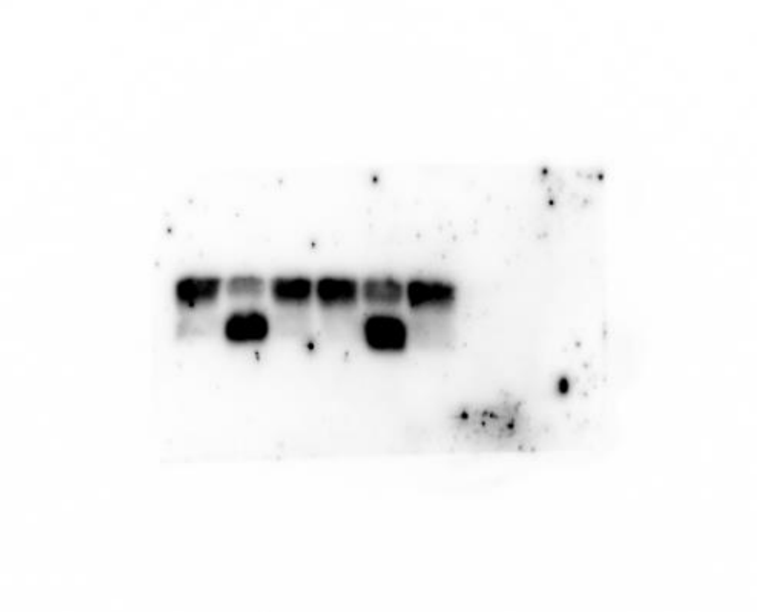

Figure5-LC3_2

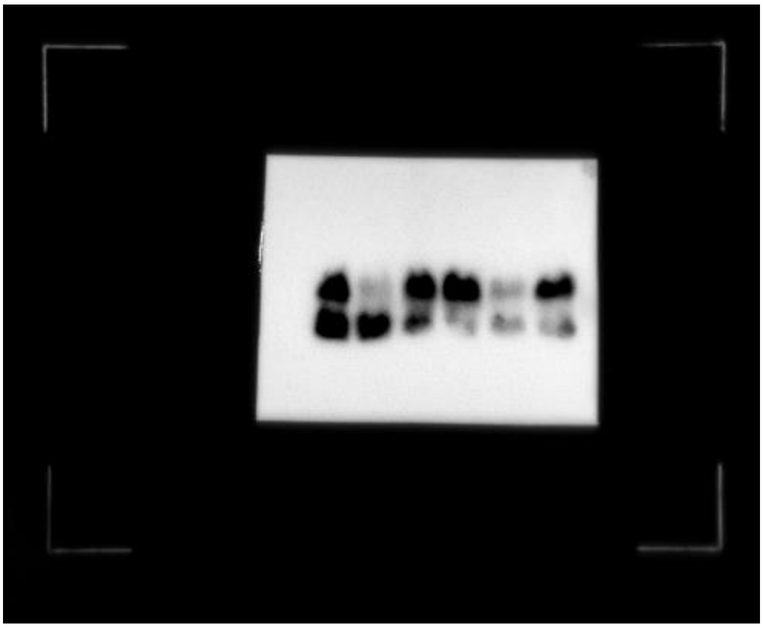

Figure5-NCAD_1

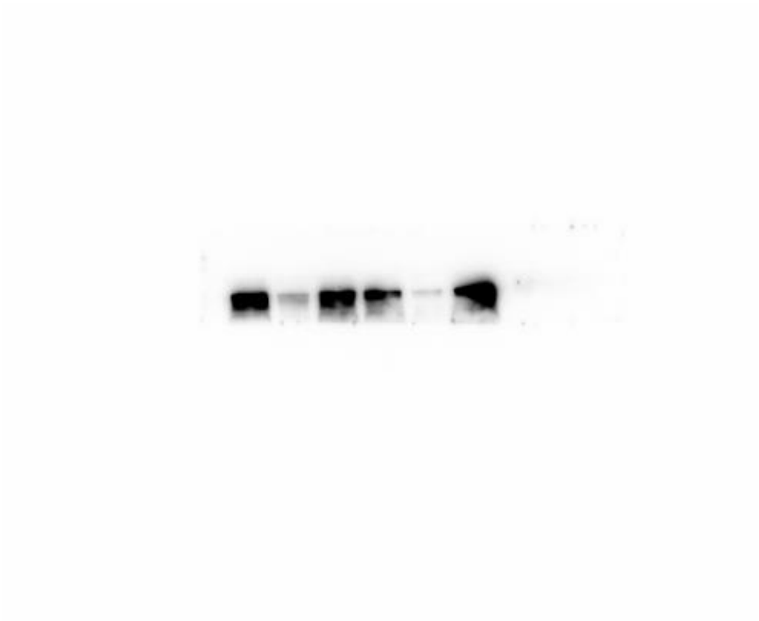

Figure5-NCAD_2

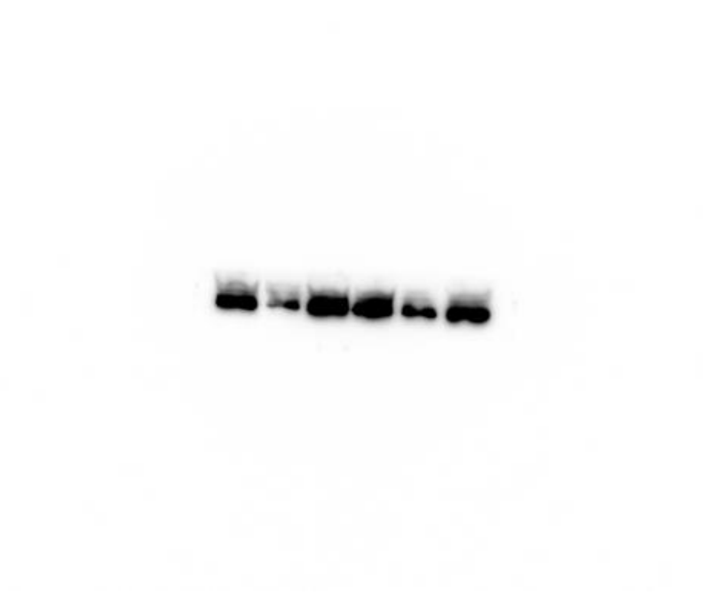

Figure5-P62_1

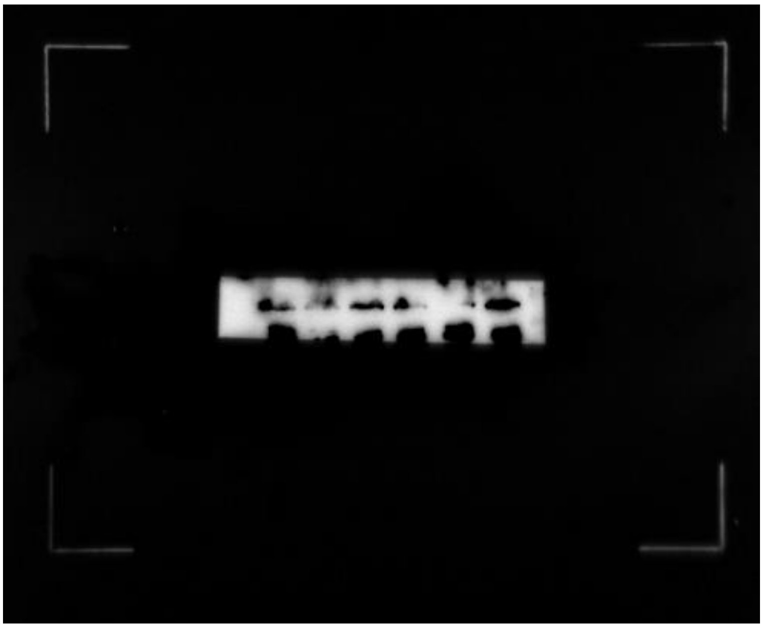

Figure5-P62_2

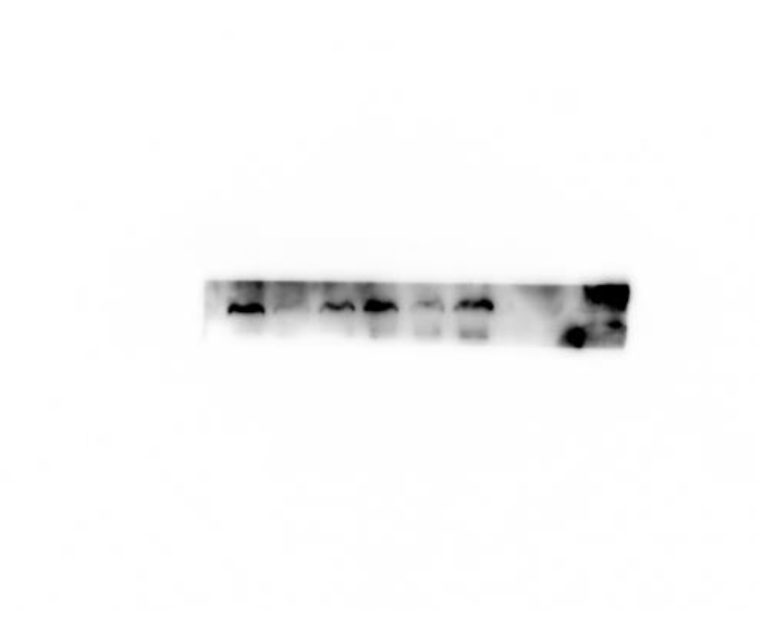

Figure5-P-ERK-1

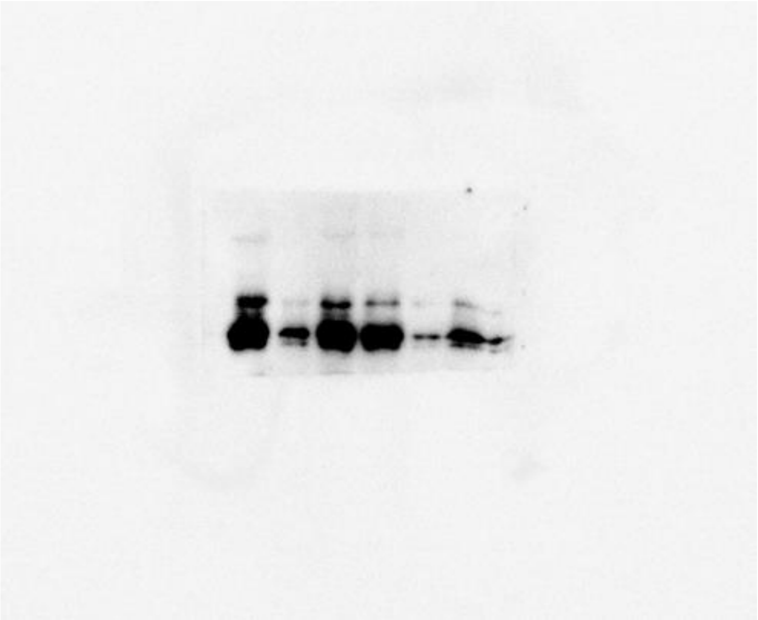

Figure5-P-ERK-2

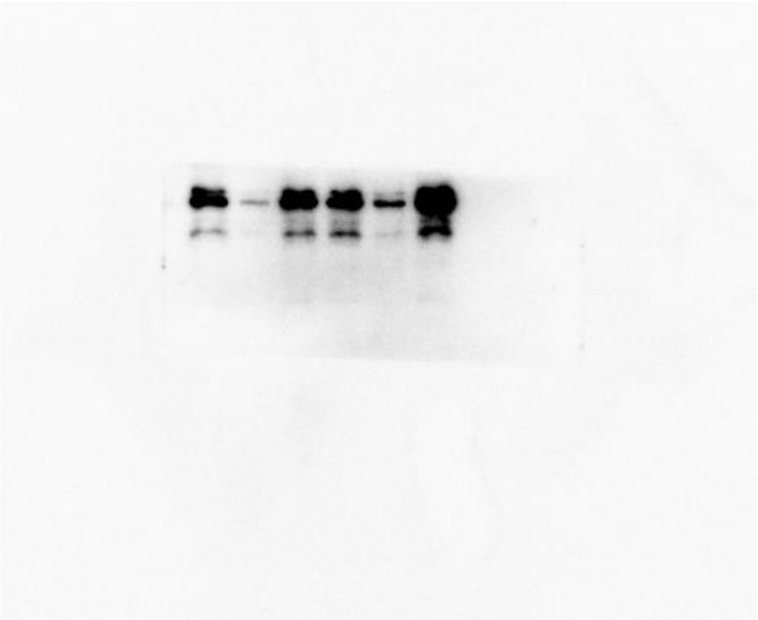

Figure5-p-JNK_1

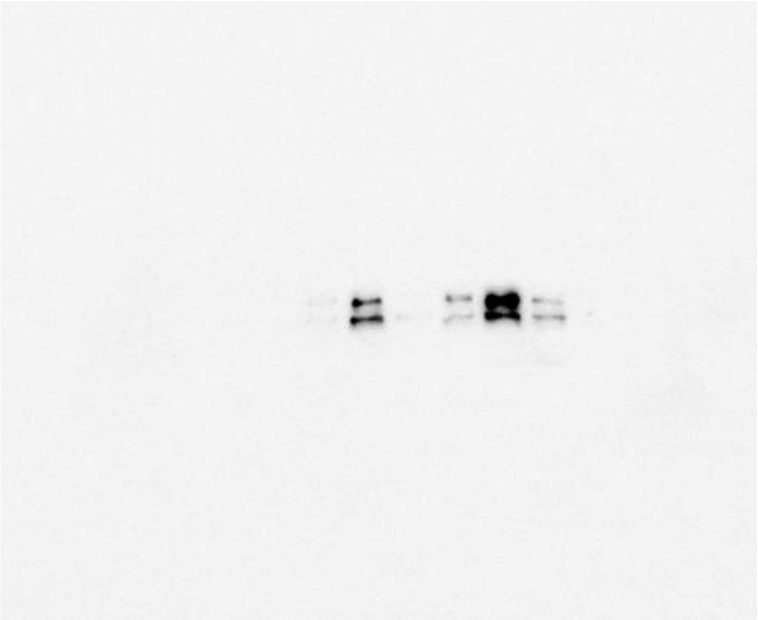

Figure5-p-JNK_2

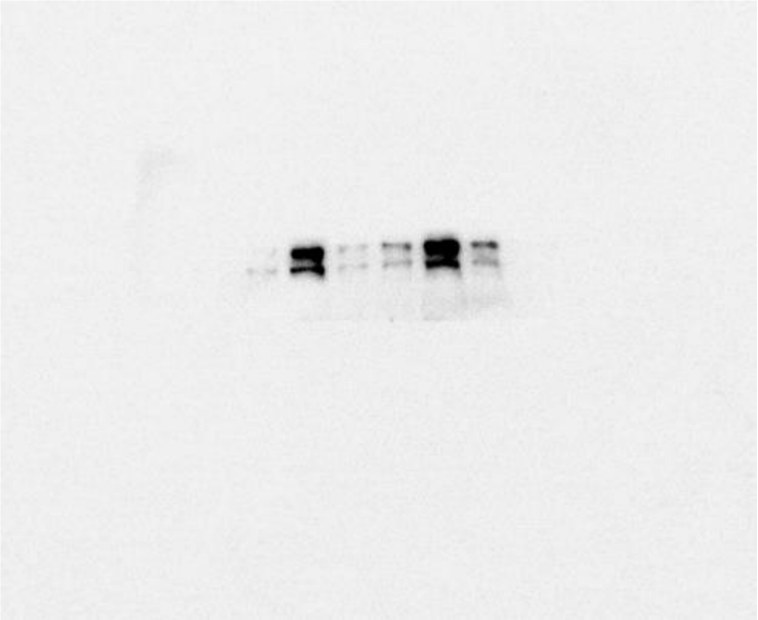

Figure5-VIMENTIN_1

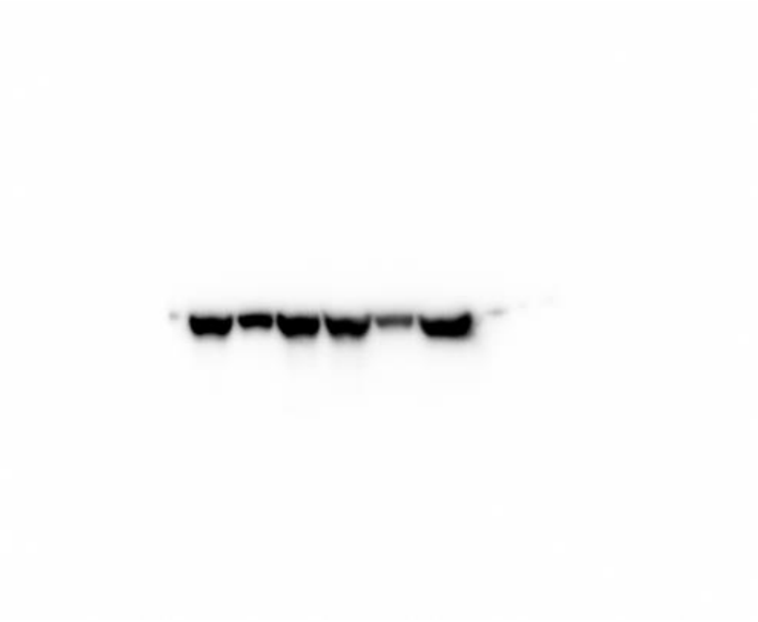

Figure5-VIMENTIN_2

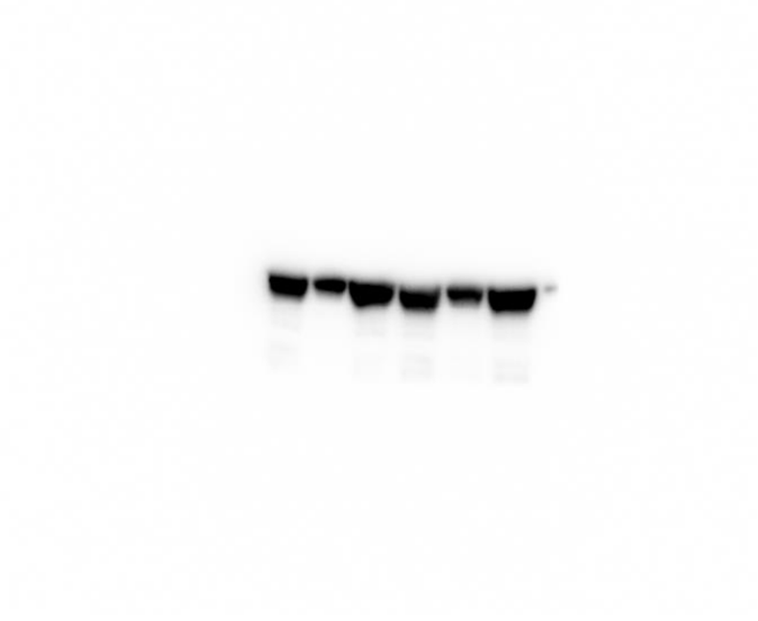

Original western blots in Figure 6

Figure6-4EBP1

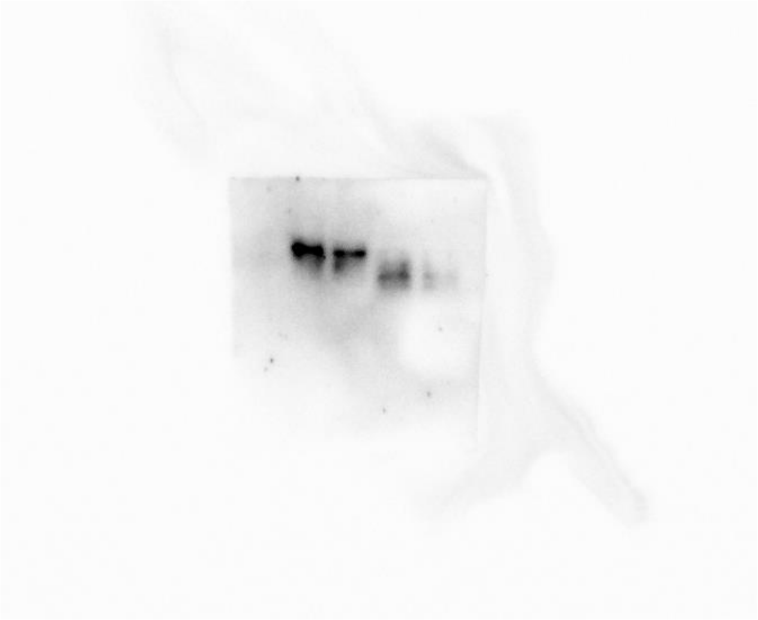

Figure6-DUSP4

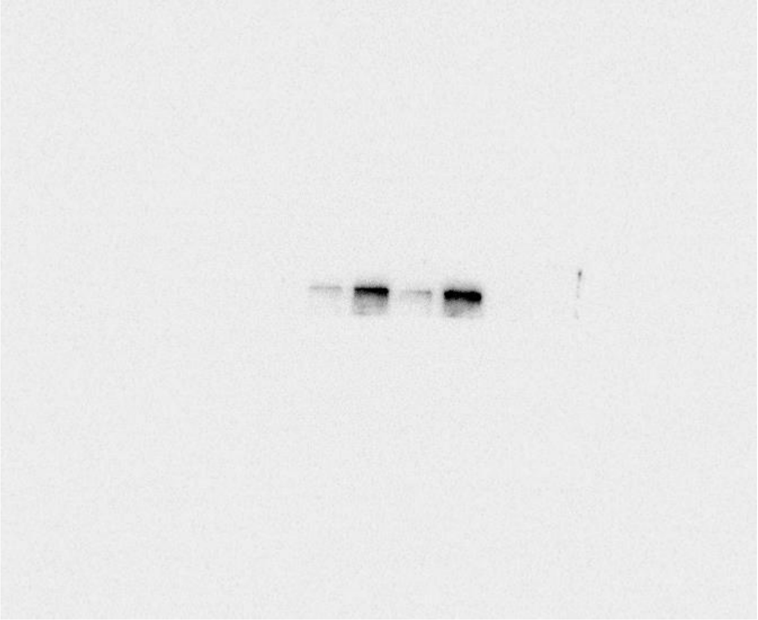

Figure6-GAPDH

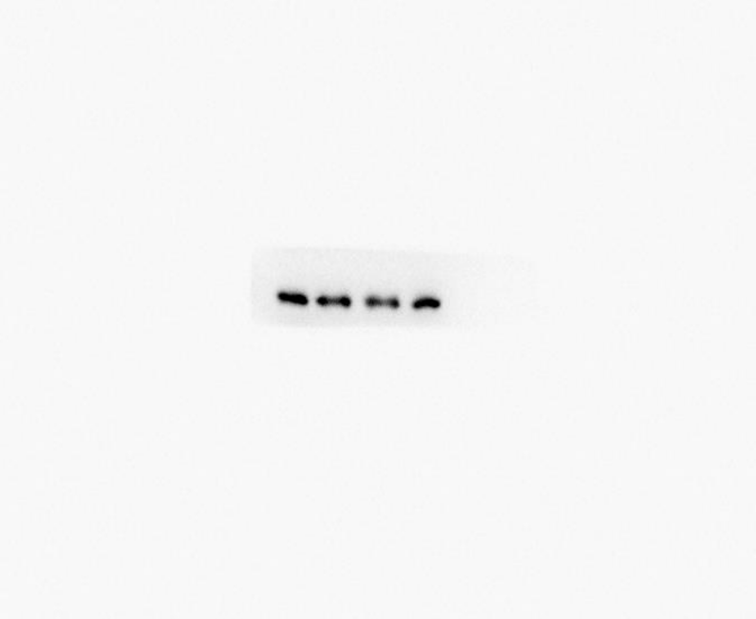

Figure6-LC3

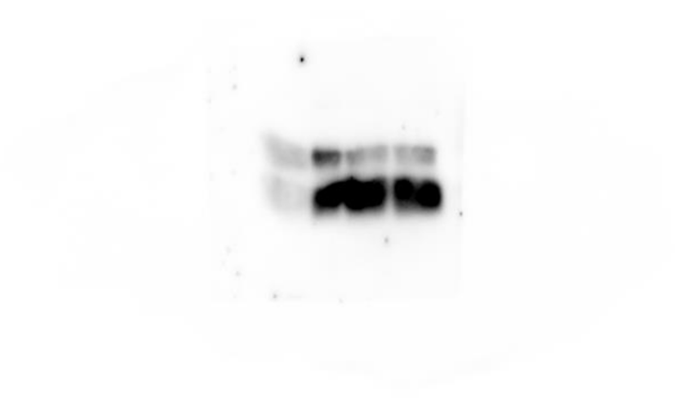

Figure6-NCAD

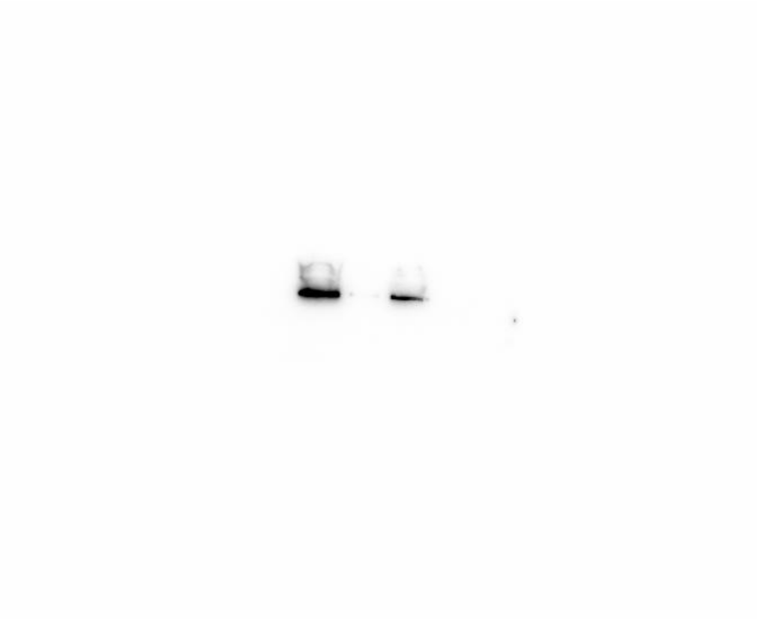

Figure6-p-4EBP1

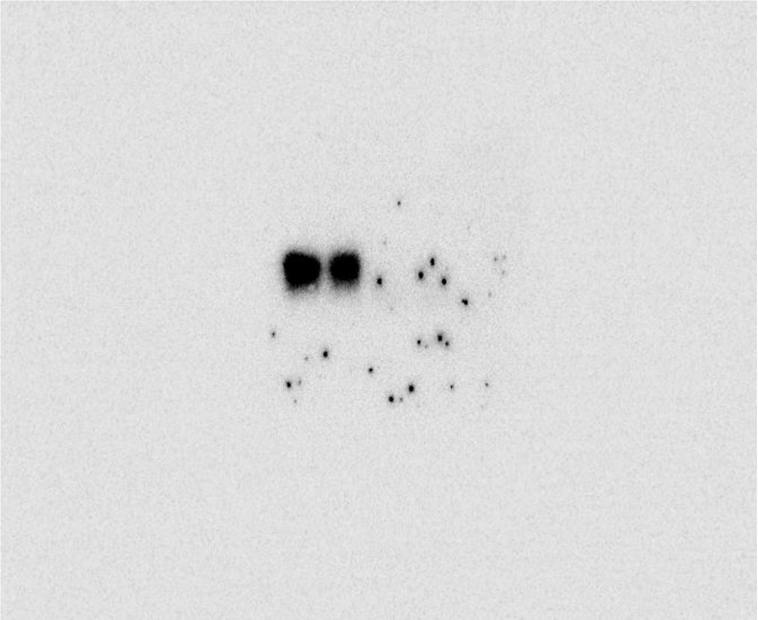

Figure6-P62

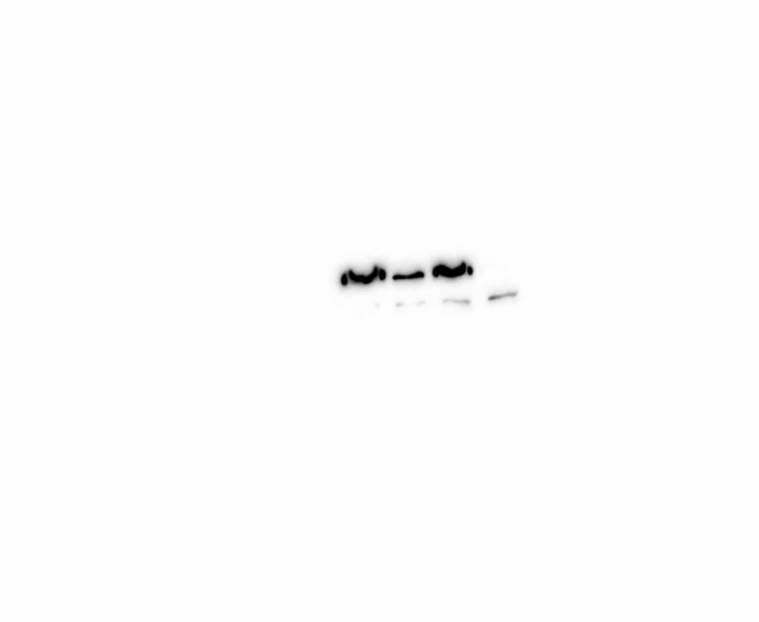

Figure6-P-ERK

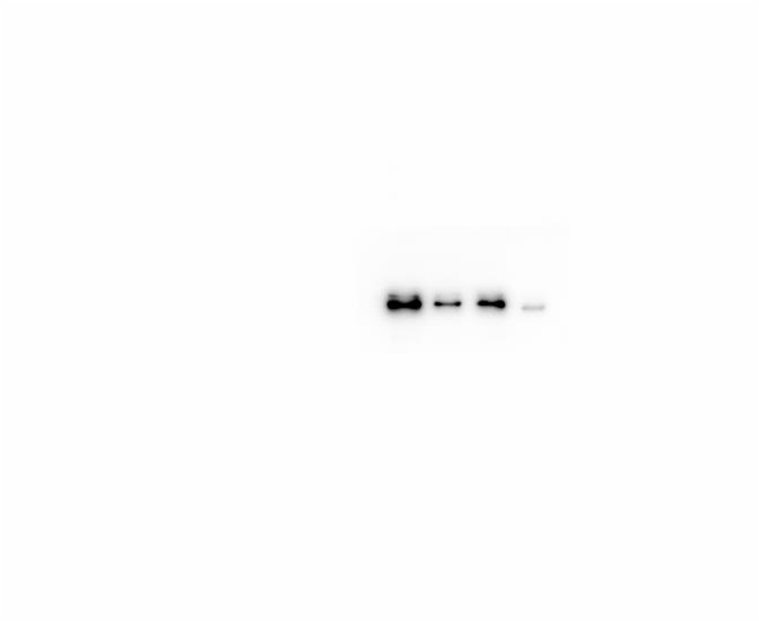

Figure6-p-JNK

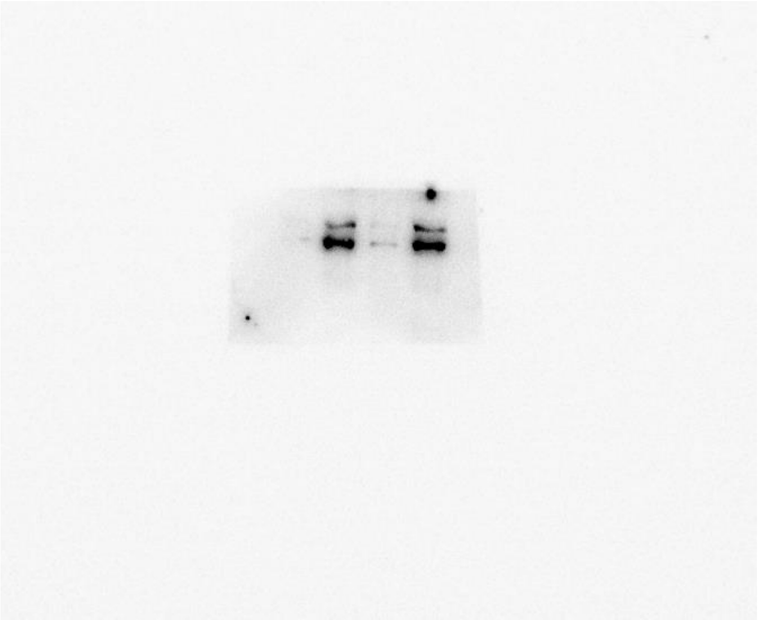

Figure6-PS6K

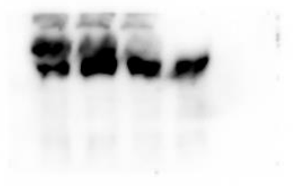

Figure6-Vimentin

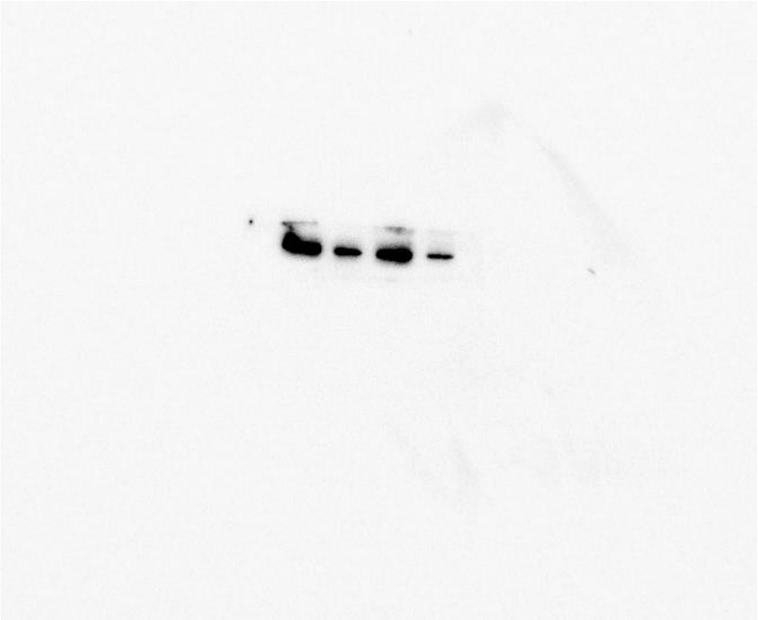

Supplement: Supplementary file 2 [file 41420_2022_1138_MOESM2_ESM.pdf]
