# Supplementary material for: Silencing circFTO inhibits malignant phenotype through modulating DUSP4 expression in clear cell renal cell carcinoma
Source: Cell Death Discov. 2022 Sep 20;8:392. doi: 10.1038/s41420-022-01138-7 (PMC9489864; doi:10.1038/s41420-022-01138-7)
Supplement: Supplementary file 3 [file 41420_2022_1138_MOESM3_ESM.pdf]

Supplementary File 3: Defferential expressed circRNAs in Bladder cancer tissue compared with normal tissue

							CPM						
**Locus**	**Gene_Name**	**Source**	**Length**	**Genomic_Length**	**Gene_ID**	**Trans_ID**	**T_CPM**	**Con_CPM**	**T1**	**T2**	**Con1**	**Con2**
**CircRNAs upregulated in tumor**												
chr1:60366636-60381772:-	CYP2J2	circBase	1120	15136	ENSG00000134716.9_2	ENST00000371204.3_1	9.969434516	3.380560492	0	1927.2626	0	0
chr18:2890558-2892484:+	EMILIN2	circBase	1926	1926	ENSG00000132205.10_2	ENST00000254528.3_1	9.863190016	3.380560492	1900.550035	0	0	0
chr8:61707544-61714152:+	CHD7	circBase	346	6608	ENSG00000171316.11_3	ENST00000423902.6_1	9.674835167	3.380560492	670.7823653	934.43035	0	0
chr12:46633461-46637097:-	SLC38A1	circBase	330	3636	ENSG00000111371.15_3	ENST00000398637.9_1	9.497611513	3.380560492	223.5941218	1168.03794	0	0
chr4:7870348-7873807:-	AFAP1	circBase	227	3459	ENSG00000196526.10_2	ENST00000358461.6_1	9.373178726	3.380560492	1229.76767	116.803794	0	0
chr16:53907697-53968021:+	FTO	circBase	469	60324	ENSG00000140718.20_3	ENST00000636491.1_1	9.268412544	3.380560492	0	1168.03794	0	0
chr7:21654731-21659696:+	DNAH11	CIRCexplorer2	663	4965	ENSG00000105877.17_2	ENST00000620169.4_1	9.252579957	3.380560492	111.7970609	1051.23414	0	0
chr18:60206913-60217693:+	ZCCHC2	circBase	374	10780	ENSG00000141664.9_4	ENST00000269499.9_3	9.249136847	3.380560492	1117.970609	116.803794	0	0
chr8:62593526-62596747:-	ASPH	circBase	219	3221	ENSG00000198363.17_3	ENST00000379454.8_3	11.65933166	6.631691595	5813.447166	700.822763	0	170.2387451
chr10:35805450-35819171:+	CCNY	circBase	315	13721	ENSG00000108100.17_2	ENST00000374706.5_1	9.788748973	5.837677068	0	1693.65501	98.1163923	0
chr13:43528083-43544806:-	EPSTI1	circBase	375	16723	ENSG00000133106.14_3	ENST00000313640.11_1	10.19764521	6.669290305	1900.550035	467.215175	98.1163923	85.11937257
chr12:46622935-46637097:-	SLC38A1	circBase	522	14162	ENSG00000111371.15_3	ENST00000398637.9_1	11.61239805	8.17529519	2906.723583	3328.90812	392.465569	170.2387451
**CircRNAs downregulated in tumor**												
chr15:53957768-54025358:-	WDR72	circBase	1974	67590	ENSG00000166415.14_2	ENST00000396328.5_1	3.380560492	9.914668997	0	0	196.232785	1702.387451
chr15:53957768-54015105:-	WDR72	CIRCexplorer2	1809	57337	ENSG00000166415.14_2	ENST00000396328.5_1	3.380560492	9.894045825	0	0	686.814746	1191.671216
chr3:17051165-17056403:+	PLCL2	circBase	2691	5238	ENSG00000154822.17_3	ENST00000615277.4_1	3.380560492	9.728729421	0	0	392.465569	1276.790589
chr15:57753877-57754090:+	CGNL1	circBase	213	213	ENSG00000128849.10_2	ENST00000281282.5_1	3.380560492	9.537037865	0	0	784.931138	680.9549806
chr6:54185376-54219434:+	TINAG	CIRCexplorer2	895	34058	ENSG00000137251.15_2	ENST00000259782.8_1	3.380560492	9.474519988	0	0	981.163923	425.5968629
chr11:22242642-22261230:+	ANO5	circBase	698	18588	ENSG00000171714.10_2	ENST00000324559.8_1	3.380560492	9.334591373	0	0	588.698354	680.9549806
chr20:52773707-52788209:-	CYP24A1	circBase	1106	14502	ENSG00000019186.9_2	ENST00000216862.7_1	6.323359992	10.99268202	0	116.803794	1667.97867	2383.342432
chr2:39559057-39564722:-	MAP4K3	circBase	220	5665	ENSG00000011566.14_3	ENST00000263881.7_2	5.780037091	9.777078785	111.7970609	0	883.04753	851.1937257
chr7:16255690-16317851:-	ISPD	circBase	416	62161	ENSG00000214960.9_2	ENST00000407010.6_2	5.781042226	9.744132368	111.7970609	0	1275.5131	425.5968629
chr6:54191599-54219434:+	TINAG	CIRCexplorer2	741	27835	ENSG00000137251.15_2	ENST00000259782.8_1	6.314753107	10.23877676	0	116.803794	686.814746	1702.387451
chr6:51798907-51900519:-	PKHD1	CIRCexplorer2	3024	101612	ENSG00000170927.14_2	ENST00000371117.7_1	6.313061385	10.13813439	0	116.803794	1471.74588	766.0743531
chr15:57743697-57754090:+	CGNL1	circBase	600	10393	ENSG00000128849.10_2	ENST00000281282.5_1	7.897208944	11.60053208	0	408.813278	2354.79341	3830.371766
chr2:207144263-207162097:+	ZDBF2	circBase	290	17834	ENSG00000204186.7_2	ENST00000374423.7_1	6.310354388	10.00212416	0	116.803794	490.581961	1532.148706
chr13:33109905-33111164:-	N4BP2L2	circBase	1259	1259	ENSG00000244754.8_3	ENST00000505213.5_2	6.695032482	10.34401589	223.5941218	0	784.931138	1787.506824
chr15:57730182-57754090:+	CGNL1	circBase	2418	23908	ENSG00000128849.10_2	ENST00000281282.5_1	7.458448991	11.05195159	0	292.009485	1667.97867	2553.581177
chr2:210968827-211019335:-	C2orf67	circBase	1457	50508	ENSG00000144445.16_2	ENST00000281772.13_2	7.904298246	11.12079441	223.5941218	233.607588	2649.14259	1787.506824
chr15:57730182-57734676:+	CGNL1	circBase	1818	4494	ENSG00000128849.10_2	ENST00000281282.5_1	8.224439415	11.3705412	0	525.617072	1864.21145	3404.774903
chr4:187627716-187630999:-	FAT1	circBase	3283	3283	ENSG00000083857.13_3	ENST00000441802.6_1	8.873969564	11.62088743	111.7970609	759.22466	3728.42291	2553.581177
chr6:51875106-51900519:-	PKHD1	CIRCexplorer2	2654	25413	ENSG00000170927.14_2	ENST00000371117.7_1	9.705706649	12.22720303	0	1576.85122	3434.07373	6128.594825
